# Stark Decline in Journalists’ Use of Preprints Postpandemic

**DOI:** 10.1177/10755470241285405

**Published:** 2024-10-11

**Authors:** Juan Pablo Alperin, Kenneth Shores, Alice Fleerackers, Natascha Chtena

**Affiliations:** 1 Simon Fraser University, Vancouver, British Columbia, Canada; 2 University of Delaware, Newark, USA; 3 The University of British Columbia, Vancouver, Canada

**Keywords:** altmetrics, COVID-19, journalism, news, preprints, science communication

## Abstract

The COVID-19 pandemic accelerated the use of preprints, aiding rapid research dissemination but also facilitating the spread of misinformation. This study analyzes media coverage of preprints from 2014 to 2023, revealing a significant postpandemic decline. Our findings suggest that heightened awareness of the risks associated with preprints has led to more cautious media practices. While the decline in preprint coverage may mitigate concerns about premature media exposure, it also raises questions about the future role of preprints in science communication, especially during emergencies. Balanced policies based on up-to-date evidence are needed to address this shift.

## Introduction

Preprints offer an exciting opportunity to sidestep many of the challenges of traditional academic publishing—namely, they offer rapid dissemination of research and lower barriers for readers and authors. However, they also present a risk—that the dissemination of unreviewed science could lead to the spread of harmful mis- and disinformation. The COVID-19 pandemic vividly underscored both sides of preprints.

Preprints were a driving force in the pandemic response, with both researchers and journalists using them to share emerging evidence at levels not seen before (Fleerackers et al., 2024; Fraser et al., 2021). Much of the media coverage of COVID-19 preprints was helpful, with some of the most widely reported preprints covering topics such as COVID-19’s aerosol and surface stability and the effectiveness of social distancing and other interventions (Fraser et al., 2021). The rise in preprints narrowed the gap between researchers, policymakers, and the public, providing unhampered access to research material with direct social relevance when it was sorely needed.

Yet, a handful of problematic preprints posted during the pandemic fueled misinformation and conspiracy theories about the SARS-CoV-2 virus. One such preprint—posted on bioRxiv on January 31, 2020, and withdrawn only 2 days later—claimed to have identified similarities between the DNA of the novel coronavirus and that of HIV and suggested that COVID-19 might therefore have been engineered by humans (Davey, 2020). Although the academic community was quick to challenge these claims, the findings had already been widely disseminated in mainstream and alternative media, as well as on discussion boards, in podcasts, and on social media.

The misinformation that spread through these high-profile cases brought new urgency to concerns about the public risks of preprints. Scientists were urged to “be extremely cautious about releasing preprint results” (Roy & Edwards, 2022, p. 1), while journalists were told to “take special precautions” (Khamsi, 2020) in vetting their quality. There was a growing consensus that the scholarly community and the media needed to “do better” in communicating about these unreviewed studies with the public (Caulfield et al., 2021).

Previous research shows that media coverage of COVID-related preprints surged during the early pandemic, while coverage of preprints on other topics saw a slight decline (Fleerackers et al., 2024). However, now that the emergency phase of the pandemic has passed, it is uncertain to what extent journalists will continue to use preprints. We suggest three hypotheses:

*Hypothesis 1*: That the pandemic taught journalists about the value of preprints and that this resulted in a modest increase in preprint use moving forward.
*Hypothesis 2*: That COVID-19 was unique and that preprint use returned to pre-COVID-19 use rates.
*Hypothesis 3*: That preprint use during the pandemic chastened journalists and alarmed them to the dangers of unvetted scholarship, resulting in a decrease in preprint use.

This study tests these hypotheses by analyzing the number of mentions of preprints in the media between January 1, 2014, and December 31, 2023, relative to the mentions of research published in journals indexed in the Web of Science (WoS).

## Materials and Methods

This study utilizes data from Altmetric collected in January 2024 and augments it with article and preprint metadata from Crossref and arXiv. We focused on the 92 media outlets that had mentioned WoS research at least 100 times every year between 2014 and 2023 (Fleerackers et al., 2024). After filtering and cleaning, our final preprint sample comprised 61,115 mentions of 21,490 preprints in 48,472 stories published by the 92 outlets in our sample (a preprint could be covered in several stories and a single story could mention multiple preprints). The final WoS-indexed research sample comprised 2,325,207 mentions of 632,815 research outputs across 1,542,571 stories published by the same 92 outlets.

To analyze the proportion of research-based media coverage that mentions preprints, we estimated multivariate fractional regressions. We estimated counts of preprints and counts of WoS publications mentioned by the media as Poisson regressions. Further details of our methods can be found in the supplementary materials.

## Results and Conclusion

**Figure 1.**
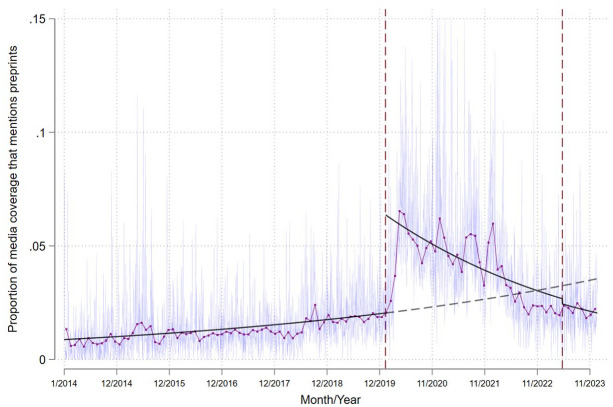
Proportion of Research-Based Media Coverage That Mentions Preprints Per Day. *Note*. Figure shows the proportion of research-based media coverage that mentions preprints per day beginning January 1, 2014, and ending December 31, 2023. The thin blue line plots the daily proportion of research-based media coverage that mentions preprints (i.e., mentions of preprints relative to mentions of preprints and WoS publications). The thicker purple line with marker symbols plots the average monthly proportion of research-based media coverage that mentions preprints, with each marker symbol plotted on the 15^th^ day of each month. The solid gray line is the predicted proportion of research-based media coverage that mentions preprints based on a fractional logistic regression that models time (in days) linearly, an intercept shift for when the COVID-19 pandemic begins, an interaction between linear time and the pandemic era, and an intercept shift for when the pandemic ends. The dashed gray line represents the predicted proportion of research-based media coverage that mentions preprints based on the trend in preprint mentions prior to the onset of the COVID-19 pandemic. Vertical lines mark the start and end of the COVID-19 pandemic according to the World Health Organization.

**Figure 2.**
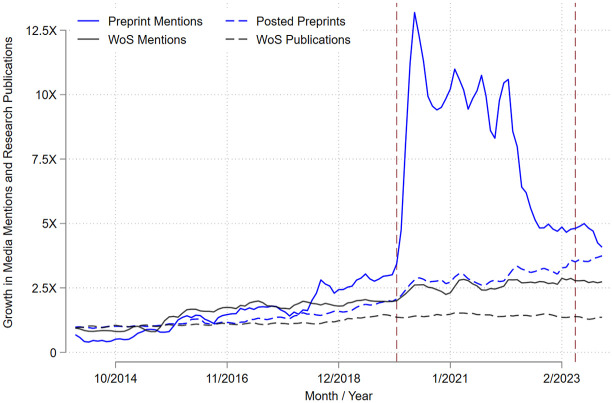
Growth Rates of Total Preprint Mentions, Total Preprints Posted, Total WoS Mentions, and Total WoS Publications. *Note*. Growth rates are calculated from January of 2014 for all nine preprint servers and for the WoS. Total number of preprint mentions, preprints posted, WoS mentions, and WoS publications are calculated for each year-month; the growth rate is the ratio of the current count in the year-month relative to the count in January 2014. Vertical lines mark the start and end of the COVID-19 pandemic according to the World Health Organization.

Our analysis provides support for the third hypothesis: following the end of the COVID-19 pandemic, media coverage of preprints has declined well below rates forecasted by prepandemic trends. This decline can be seen both in the proportion of research-based media coverage that mentions preprints (Figure 1) and in absolute numbers (Figure 2), following the initial surge during the pandemic’s early stages. We calculate that, one year into the pandemic, the share of research-based media coverage mentioning preprints had increased by 2.1 percentage points (PP; *p* < .000; 95% CI [2.6–1.6]; predicted share of preprint mentions on January 10, 2021, based on prepandemic trends equal to 2.4 PP; observed share of preprint mentions equal to 4.5 PP). By the end of 2023, it had decreased by 1.4 PP (*p* < .000; 95% CI [0.9–2.0]; predicted share of preprint mentions on December 31, 2023, based on prepandemic trends equal to 3.5 PP; observed share of preprint mentions equal to 2.1 PP; Figure 1). Importantly, these changes in the proportion of research-based media coverage mentioning preprints were not caused by changes in journalists’ treatment of WoS publications, as media coverage of WoS publications throughout this period was largely invariant to the pandemic (Figure 2).

Across the nine preprint servers included in our analysis, by December 2023, we estimate a 68% decline in the number of preprints mentioned in the media (*p* < .000; 95% CI [0.58 – 0.78]), relative to what we would anticipate based on prepandemic trends (Table 1; predicted preprint mentions based on prepandemic trends equal to 1,473.6; observed preprint mentions equal to 473.6). The size of the decline differs by server, ranging from 77% (bioRxiv/medRxiv) to as high as 94% Social Science Research Network (SSRN). Only media coverage of arXiv preprints remains unaffected, with estimated mentions of preprints at the same level as predicted using prepandemic data. Moreover, declines in media coverage of preprints remain the same or become more pronounced when controlling for the number of preprints posted to the servers. Indeed, while the number of available preprints increased steadily and linearly throughout and after the pandemic, media mentions of preprints increased dramatically during COVID-19 and then declined.

**Table 1.** Proportional Decline in Counts of Media Mentions (One Minus the Ratio of Predicted Counts to Observed Media Mentions).

Server	[1] Unconditional	[2] Conditional
All servers	0.68*** (0.05)	0.71*** (0.08)
WoS	0.35*** (0.04)	0.35*** (0.04)
bioRxiv/medRxiv	0.77** (0.09)	0.83** (0.09)
bioRxiv	0.86*** (0.05)	0.88* (0.06)
arXiv	0.00*** (0.24)	0.04*** (0.24)
National Bureau of Economic Research (NBER)	0.89 (0.08)	0.89 (0.08)
Social Science Research Network (SSRN)	0.94*** (0.01)	0.96*** (0.01)

*Note*. This table shows the ratio of the predicted number of preprint mentions based on prepandemic trends relative to the estimated number of preprint mentions in December, 2023. Model coefficients are estimated using Poisson regression, and the predicted ratio of preprint mentions after the pandemic relative to predicted preprint mentions using prepandemic trends is generated from model coefficients. Model [1] Unconditional reports the estimated ratio without additional covariates, and Model [2] Conditional reports the estimated ratio controlling for the number of preprints posted and WoS publications each month. For the WoS result, we also control for a January-specific effect due to publications without specific publication dates being assigned to January 1 by the WoS indexing process. For the medRxiv result, we combine medRxiv mentions with bioRxiv mentions because there is insufficient medRxiv data to calculate pre-COVID-19 trends.

Significance levels: *p < 0.05, **p < 0.01, ***p < 0.001.

Notably, we also estimate a 35% decline in the number of WoS publications mentioned in the media postpandemic relative to prepandemic trends. As with preprints, this decline remains unchanged when controlling for the number of WoS publications. However, although this decline relative to the expected is substantial, it pales in comparison with the decline in media mentions of preprints, especially for the two social sciences servers (SSRN and National Bureau of Economic Research [NBER]).

This decline in preprint coverage supports findings from interviews conducted with health and science journalists in prior work (Fleerackers et al., 2022). These specialized journalists voiced concerns about the potential risks of spreading misinformation and ultimately harming their audiences. While they found vetting preprints challenging, many reported using rigorous strategies to do so: triangulating findings with those of other studies and seeking critical input from unaffiliated scientists. Most importantly, these journalists strove to put the interests of the public first, limiting their coverage of preprints to instances when they felt the potential benefits of doing so outweighed the possible risks. Combined with the results of the current analysis, there is reason to believe that other journalists may also be well-aware of the risks of covering preprints and are now taking a more cautious approach to when and how they report on them.

This reduction in media coverage may alleviate one of the chief concerns expressed in a recent global survey of attitudes toward preprinting: “premature media coverage” (Ni & Waltman, 2024). Given the timing of the survey (late 2022 and early 2023), it is likely that this concern, expressed most clearly by those in life and health sciences, was in no small part informed by the explosion of preprints that circulated in the media during the pandemic—particularly preprints that were later found to be flawed or problematic. However, our findings suggest that journalists have become more, not less, discerning about when they cover these unreviewed studies.

This change in journalists’ knowledge, attitudes, or willingness to cover preprints may alleviate concerns about the spread of misinformation, but also raises new questions that warrant reflection. Does the change in journalists’ willingness to cover preprints signal a broader transformation in their understanding or attitudes toward science and, particularly, science-in-progress? Conversely, how might the public’s knowledge and attitudes toward preprints, research, or science-based journalism change in the aftermath of the pandemic? And, perhaps most consequentially, when another emergency presents itself, will journalists, scholars, and other science communicators be able, or willing, to use preprints to rapidly circulate life-saving research in the same way?

As we set policies, develop guidelines, and establish best practices for how and when to use preprints going forward, it is essential that we recognize that journalists’ use of COVID-19 preprints was—like so many aspects of the pandemic—a trial by fire and a crucible of learning. The concerns evidenced during the pandemic period should not make us forget that it was rapid and low-barrier publishing, informal review (not formal peer review), and media coverage that made preprints so effective in a time of crisis.

## Supplemental Material

sj-docx-1-scx-10.1177_10755470241285405 – Supplemental material for Stark Decline in Journalists’ Use of Preprints Postpandemic Supplemental material, sj-docx-1-scx-10.1177_10755470241285405 for Stark Decline in Journalists’ Use of Preprints Postpandemic by Juan Pablo Alperin, Kenneth Shores, Alice Fleerackers and Natascha Chtena in Science Communication

## References

[bibr1-10755470241285405] Caulfield T. Bubela T. Kimmelman J. Ravitsky V. (2021). Let’s do better: Public representations of COVID-19 science. FACETS, 6, 403–423. 10.1139/facets-2021-0018

[bibr2-10755470241285405] Davey M. (2020, May 29). Covid-19 study on hydroxychloroquine use questioned by 120 researchers and medical professionals. The Guardian. https://www.theguardian.com/world/2020/may/29/covid-19-surgisphere-hydroxychloroquine-study-lancet-coronavirus-who-questioned-by-researchers-medical-professionals

[bibr3-10755470241285405] Fleerackers A. Moorhead L. Maggio L. A. Fagan K. Alperin J. P. (2022). Science in motion: A qualitative analysis of journalists’ use and perception of preprints. bioRxiv. 10.1101/2022.02.03.479041 PMC9678308 36409723

[bibr4-10755470241285405] Fleerackers A. Shores K. Chtena N. Alperin J. P. (2024). Unreviewed science in the news: The evolution of preprint media coverage from 2014–2021. Quantitative Science Studies, 5(2), 297–316. 10.1162/qss_a_00282

[bibr5-10755470241285405] Fraser N. Brierley L. Dey G. Polka J. K. Pálfy M. Nanni F. Coates J. A. (2021). The evolving role of preprints in the dissemination of COVID-19 research and their impact on the science communication landscape. PLOS Biology, 19(4), e3000959. 10.1371/journal.pbio.3000959 PMC8046348 33798194

[bibr6-10755470241285405] Khamsi R. (2020, June 1). Problems with preprints: Covering rough-draft manuscripts responsibly. The Open Notebook. https://www.theopennotebook.com/2020/06/01/problems-with-preprints-covering-rough-draft-manuscripts-responsibly/

[bibr7-10755470241285405] Ni R. Waltman L. (2024). To preprint or not to preprint: A global researcher survey. Journal of the Association for Information Science and Technology, 75(6), 749–766. 10.1002/asi.24880

[bibr8-10755470241285405] Roy S. Edwards M. A. (2022). Addressing the preprint dilemma. International Journal of Hygiene and Environmental Health, 240, 113896. 10.1016/j.ijheh.2021.113896 34864599

