# Supplementary material for: Stark Decline in Journalists’ Use of Preprints Postpandemic
Source: Sci Commun. 2024 Oct 11;47(6):897–905. doi: 10.1177/10755470241285405 (PMC12539774; doi:10.1177/10755470241285405)
Supplement: sj-docx-1-scx-10.1177_10755470241285405 – Supplemental material for Stark Decline in Journalists’ Use of Preprints Postpandemic [file sj-docx-1-scx-10.1177_10755470241285405.docx]

Detailed Methods for:

Stark Decline in Journalists’ Use of Preprints Postpandemic

Alperin, J.P., Shores, K., Fleerackers, A., Chtena, N. (2024). Stark Decline in Journalists’ Use of Preprints Post-pandemic. *Science Communication*.

**I. Data Collection**

We collected mentions of research in the news media using Altmetric, a company tracking mentions of research outputs across various digital media platforms. The Altmetric “Mainstream Media” category has been shown to be reliable for identifying research mentions across a predetermined list of English-language media sources (1). As such, we used a list of 94 English-language outlets curated for an earlier study (2) that had mentioned research at least 100 times every year between 2014 and 2020. To continue with the same criteria, we removed two outlets that did not meet this 100-article threshold between 2021 and 2023, leaving us with a list of 92 outlets covering a wide range of topics (e.g., science/technology, health/medicine, business, general news, etc). By querying the Altmetric Explorer API in the first week of 2024, we downloaded details of every time any research (including preprints and Web of Science [WoS] research outputs) was mentioned in these outlets.

We subsequently queried the Crossref API to collect metadata details for every DOI that had been mentioned in the 92 media outlets and the arXiv API for every arXiv ID mentioned. Using this metadata, we identified which mentions belonged to the following preprint servers: arXiv, bioRxiv, chemRxiv, medRxiv, NBER, OSF, Research Square, SSRN, and techRxiv. We compared the ISSN found in the Crossref metadata with a list of the ISSNs of every journal in the WoS to identify which mentions were associated with journals indexed in that database. For simplicity, we refer to these documents published in journals that are indexed in the Web of Science as “WoS publications” and to mentions of these documents as “WoS mentions.”

Following the same approach as Fleerackers et al. (2024), we determined the most likely publication date for each preprint or WoS publication. For arXiv, it was the date provided by the arXiv API; for SSRN, it was either the “first posted on” date provided by Altmetric or Crossref’s DOI creation date, whichever came first, and for all other servers, it was the DOI creation date.

Finally, between February and April 2024, we collected the number of preprints posted each month for all the servers in our dataset. We collected the counts for arXiv, bioRxiv, chemRxiv, medRxiv, OSF, Research Square, and techRxiv by querying the Dimensions database. The number of preprints posted on NBER each month were not available from Dimensions, so we downloaded the metadata for every preprint in the IDEAS/RePEc repository and calculated counts based on their publication dates. Because preprint counts for SSRN reported in Dimensions showed large volatility, we downloaded monthly preprint counts by scraping reports made available on the SSRN homepage. Finally, we collected the number of documents found in WoS by querying the WoS using the web interface and filtering for one month at a time using the date of publication (DOP) filter.

**II. Data Cleaning**

We identified 71,891 mentions of preprints across the 92 outlets. From these, we filtered out 8,081 mentions of preprints with publication dates before 2013, another 641 mentions of preprints whose publication dates suggested they were postprints rather than preprints (i.e., the preprint date was less than a week before, or was after, the corresponding paper’s publication date), and an additional 327 mentions where the preprint publication date was after the date of the mention (i.e., could not have been possible and must therefore be caused by a metadata error). Finally, we removed 1,727 duplicate mentions. In total, filtering led to the exclusion of 10,776 mentions (15.0% of original dataset). Our final preprint sample comprised 61,115 mentions of 21,490 preprints in 48,472 stories published by the 92 outlets in our sample.

We identified 2,708,008 WoS mentions across the 92 outlets. From these, we filtered out 350,884 mentions with publication dates before 2013 and removed 31,917 duplicate mentions. In total, filtering led to the exclusion of 382,801 mentions (14.1% of original dataset). The final WoS sample comprised 2,325,207 mentions of 632,815 distinct research outputs across 1,542,571 stories published by the 92 outlets in our sample.

**III. Statistical Methods**

We employ a fractional logistic regression model to examine the proportion of research-based media coverage that mentions preprints (i.e., the number of mentions of preprints relative to mentions of preprints and WoS outputs; *dailyshare*). The dependent variable, *dailyshare*, is bounded between 0 and 1 and represents the proportion of media mentions of research that are from preprint servers on day *t*. The primary explanatory variables include the days where media mentions are tabulated beginning on January 1, 2014 and ending December 31, 2023 (*groupdaily*), the days where media mentions are tabulated beginning on Jan 10, 2020 when COVID-19 was declared a pandemic by the World Health Organization (*timesince)*, an indicator variable for the start of the COVID-19 pandemic on that date (*covidstarts*), an indicator variable for the end of the COVID-19 pandemic (*covidends*), and an interaction between *timesince* and *covidstarts*. Because the time-period after *covidends* is short we do not include a separate interaction between *groupdaily* and this variable. The fractional logistic regression model estimates the log-odds of the dependent variable, which is then transformed to probabilities using the logistic function. Statistical analysis was performed using Stata version 17 (StataCorp, 2021).

The fractional logistic regression model can be specified as follows:

$$logit\left( dailyshare \right)_{t}=\beta_{0}+\beta_{1}Groupdaily+\beta_{2}Covidstarts+\beta_{3}Timesince*Covidstarts+\beta_{4}Covidends+\varepsilon_{t}$$

where:

- $logit\left( dailyshare \right)_{it}$ is the log-odds of the proportion of research-based media coverage that mentions preprints for day *t*.
- ​$\beta_{0}$ is the intercept.
- $\beta_{1}$is the coefficient for the number of daily media mentions.
- $\beta_{2}$is the coefficient for the start of the COVID-19 pandemic.
- $\beta_{3}$​is the coefficient for the interaction term between daily media mentions beginning with the COVID-19 pandemic and the start of the COVID-19 pandemic.
- $\beta_{4}$ is the coefficient for the end of the COVID-19 pandemic.
- $\varepsilon_{t}$ is the error term and is robust to heteroskedasticity.

We then calculate the marginal change in predicted proportions of research-based media coverage that mentions preprints one year into the COVID-19 pandemic (January 10, 2021) due to the COVID-19 effect, i.e., the average change in predicted proportions of media mentions focused on preprints one-year into the pandemic. We then calculate the predicted change in the proportion of research-based media coverage that mentions preprints after the pandemic relative to the predicted proportion of research-based media coverage that mentions preprints based on pre-pandemic trends. We conduct these tests by transforming model coefficients from log-odds to predicted proportions and plugging in known values of the regressors.

Specifically, we conducted a post-estimation test using the nonlinear combination of estimators (nlcom) command in Stata. This test compared the predicted counts for two scenarios:

1. **Scenario 1**: Predicted count of mentions on January 10, 2021, based solely on pre-pandemic trends (i.e., setting COVID-19-related variables to zero).
2. **Scenario 2**: Predicted count of mentions on January 10, 2021, one year into the pandemic, including the effects of all variables, especially the main effect of $\beta_{2}$, which represents the change in the share of preprints mentioned by the media when the pandemic began.

These scenarios can then be estimated as the predicted proportion of research-based media coverage that mentions preprints one-year into the pandemic using pre-pandemic trends as:

$\hat{P}_{1}=\frac{1}{1+exp(-(\beta_{0}+\beta_{1}*2,566))}$, where 2,566 corresponds to the date January 10, 2021 in our sample

$\hat{P}_{2}=\frac{1}{1+exp(-{(\beta}_{0}+\beta_{1}*2,566+\beta_{2}+\beta_{3}*366))}$, which captures the predicted change in the share of preprint media mentions on January 10, 2021 and includes the estimated pandemic effect and the linear change in preprint mentions 366 days into the COVID-19 pandemic. The estimated change in preprint mentions is the difference between $\hat{P}_{1}$and $\hat{P}_{2}$, which we estimated using the nlcom command in Stata.

We conduct a similar test to estimate the change in share of preprint mentions after the pandemic, substituting 3,630 for 2,566 and 1,430 for 366, which corresponds to the date December 10, 2023 in our sample, and including the effect of $\beta_{4}$when calculating $\hat{P}_{2}$.

**Figure 2.**

Figure 2 is based on a collapsed dataset of counts of preprint mentions, posted preprints, WoS mentions, and WoS publications. This collapsed data is available at the server level (e.g., with counts for NBER and arXiv individuated) and pooled across all servers. Figure 1 represents the growth rate in mentions and publications for all preprint servers and WoS separately. The growth rate is calculated as the observed mention/publication count relative to the mention/publication count in January 2014, the month-year our data begins. WoS publications are sometimes reported annually and assigned a January publication date, resulting in an apparent surge in publications in January of each year. To allow for annual secular growth in publications but remove the January-specific effect, we calculate the average annual publication count for WoS excluding January and then take the difference between the January publication count and the average annual publication count minus January. This difference represents the “January effect”; we then remove the January effect from January and divide that effect by 12, distributing it across all months in the year. Lastly, we apply a simple moving average smoother that replaced the observed mention/publication with the average of the preceding, observed, and subsequent month (i.e., a +/-1 smoother). No other modifications to the data were made.

**Table 1.**

To generate the reported ratios of estimated preprint mentions relative to predicted preprint mentions based on pre-pandemic trends, we employed a Poisson regression model to estimate the number of media mentions in a given month-year. The model accounts for various factors, including the introduction of the medRxiv server, the start and end of the COVID-19 pandemic, and two time trends, one for the pre-pandemic period and another for the pandemic period. We do not include an interaction between the post-pandemic period and months because the time-period is so short. When we estimate counts for all servers, we collapse the data to obtain the total count of preprint research mentioned by the media; for individual servers, we use the server-specific count. The model is specified as follows:

Equation (2)

$$\log\left( \lambda_{it} \right)=\beta_{0}+\beta_{1}Medrxiv+ \beta_{2}TimeCent+\beta_{3}CovidStarts+\beta_{4}TimeSince+ \beta_{5}CovidEnds+\varepsilon_{it}$$

Where:

- ​$\lambda_{it}$ is the expected count of media mentions for preprint/publication *i* posted on month *t.*
- $\beta_{0}$​ is the intercept.
- $\beta_{1}$​ represents the effect of the medRxiv server coming online (*Medrxiv* is a binary variable).
- $\beta_{2}$​ represents the effect of time since the start of the pandemic (*TimeCent* is a continuous variable centered at the start of the pandemic).
- $\beta_{3}$​ represents the effect of the COVID-19 pandemic starting (*CovidStarts* is a binary variable).
- $\beta_{4}$​ represents the effect of time since the start of the COVID-19 pandemic (*TimeSince* is a continuous variable coded as 0 prior to the pandemic and then set equal to 1 and increasing incrementally by 1 for each month beginning with the start of the COVID-19 pandemic).
- $\beta_{5}$represents the effect of the COVID-19 pandemic ending (*CovidEnds* is a binary variable).
- $\varepsilon_{it}$ is the error term and is robust to heteroskedasticity.

Following the estimation of the Poisson regression model, we conducted a post-estimation test using the nonlinear combination of estimators (nlcom) command in Stata. This test compared the predicted counts for two scenarios:

1. **Scenario 1**: Predicted count of mentions 48 months after the start of the pandemic, which corresponds to December 2023, including the effects of all variables.
2. **Scenario 2**: Predicted count of mentions 48 months after the start of the pandemic, based solely on pre-pandemic trends (i.e., setting COVID-19-related variables to zero).

Let $\lambda_{1}$ represent the predicted count of mentions for Scenario 1 and $\lambda_{2}$​ represent the predicted count of mentions for Scenario 2.

Predicted Count for Scenario 1:

$\log\left( \lambda_{it} \right)=\beta_{0}+\beta_{1}+ \beta_{2}*48+\beta_{3}+\beta_{4}*47+ \beta_{5}$; where 47 represents the number of months after the first month of the COVID-19 pandemic.

Predicted Count for Scenario 2:

$$\log\left( \lambda_{it} \right)=\beta_{0}+\beta_{1}+ \beta_{2}*48$$

The nonlinear combination of these estimates provides the ratio of the predicted counts; we subtract this ratio from 1 to represent the decline in preprint mentions:

$$\frac{\hat{\lambda_{1}}}{\hat{\lambda_{2}}}=1-\left[ \frac{\exp(\beta_{0}+\beta_{1}Medrxiv+ \beta_{2}TimeCent*48+\beta_{3}CovidStarts+\beta_{4}TimeSince*47+ \beta_{5}CovidEnds )}{exp(\beta_{0}+\beta_{1}Medrxiv+ \beta_{2}TimeCent*48)} \right]$$

This equation then then simplifies to

$$\frac{\hat{\lambda_{1}}}{\hat{\lambda_{2}}}= 1-exp(\beta_{3}CovidStarts+\beta_{4}TimeSince*47+ \beta_{5}CovidEnds )$$

In the conditional models, we also include counts of preprints/publications (total across servers, for WoS, and individually by preprint server, in separate regressions) as control variables. For regressions for WoS, we include an additional binary variable for the month of January to account for the fact that, for journals that do not provide month of publication, the WoS indexing approach assign the entire year’s publications to January.

**References**

- 1. J. L. Ortega, Altmetrics data providers: A meta-analysis review of the coverage of metrics and publication. *El Profesional de La Información*, 29 (2020). <https://doi.org/10.3145/epi.2020.ene.07>
  2. A. Fleerackers, K. Shores, N. Chtena, J.P. Alperin, Unreviewed science in the news: The evolution of preprint media coverage from 2014–2021. Quant. Sci. Stud. 5, 297–316 (2024). <https://doi.org/10.1162/qss_a_00282>
